# Intrathoracic Extramedullary Hematopoiesis Arising in the Anterior Mediastinum

**DOI:** 10.1002/ccr3.72605

**Published:** 2026-05-26

**Authors:** Yoshiki Kozu, Tomoaki Kinno, Kazunori Kajino, Kenji Suzuki

**Affiliations:** ^1^ Department of General Thoracic Surgery Juntendo Tokyo Koto Geriatric Medical Center Tokyo Japan; ^2^ Department of Diagnostic Pathology Juntendo Tokyo Koto Geriatric Medical Center Tokyo Japan; ^3^ Department of General Thoracic Surgery Juntendo University School of Medicine Tokyo Japan

**Keywords:** differential diagnosis, extramedullary hematopoiesis, mediastinal disease, thoracotomy

## Abstract

Anterior mediastinal extramedullary hematopoiesis (EMH) is rare and can mimic thymic tumors. Slow growth rate and evolving fat content should prompt inclusion of EMH in the differential diagnosis and caution regarding biopsy/invasive procedures in hypervascular lesions. Histologic confirmation is desirable to obtain a definitive diagnosis and guide management.

## Graphical abstract

Arrows indicate enlarging anterior (a, b) and posterior (c, d) mediastinal extramedullary hematopoiesis (EMH). EMH should be included in the differential diagnosis of anterior mediastinal lesion. Histologic confirmation is desirable to obtain definitive diagnosis and guide management.

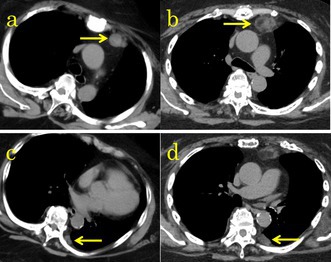

## 1 Introduction

Extramedullary hematopoiesis (EMH) is defined as hematopoiesis occurring outside the bone marrow, serving as a compensatory mechanism for inadequate bone marrow function or ineffective erythropoiesis [1]. EMH is typically associated with hematologic disorders such as myelofibrosis, lymphoma, leukemia, thalassemia, hereditary spherocytosis, sickle cell anemia, and chronic anemia. Most patients with EMH are asymptomatic, and as a result, it is often detected incidentally during routine medical checkups.

EMH can involve almost all body sites, including thoracic structures. Intrathoracic EMH is most commonly found in the posterior mediastinum, where it presents as paravertebral masses [1]. However, other manifestations of intrathoracic EMH have been sporadically reported. Here, we report a very rare case of the anterior mediastinal EMH (third described) occurring without any underlying hematological disorders.

## 2 Case History/Examination

A 67‐year‐old obese woman (body mass index: 34.0) was referred to our department due to enlarging mediastinal lesions. Her medical history included systemic lupus erythematosus, managed with 5 mg of prednisolone daily, ossification of the posterior longitudinal ligament, and a thoracic compression fracture. Due to these comorbidities, she was wheelchair‐bound. She had no history of hematological disorders and remained asymptomatic, with no signs of hepatosplenomegaly. Her complete blood count was within normal limits: white blood cell count of 5700/μL with a normal differential, hemoglobin of 14.3 g/dL, and a platelet count of 278 × 10^9^/L. Other laboratory findings were also unremarkable.

A chest X‐ray revealed a slight prominence of the superior mediastinum, projecting to the left (Figure 1). Unenhanced chest computed tomography (CT) showed a well‐demarcated anterior mediastinal lesion located behind the sternum at the left second costochondral junction, which had increased in size from 2.6 cm to 3.7 cm over 4 years (Figure 2a,b). The CT attenuation pattern within the lesion had also changed over time, shifting from a predominantly soft tissue density to a heterogeneous composition of soft tissue (30 to 40 Hounsfield unit) and fat (−80 to −60 Hounsfield unit). Additionally, a small lesion was detected in the posterior mediastinum at the left seventh intercostal space, which had enlarged from 1.5 cm to 2.0 cm over the same period (Figure 2c,d). The CT attenuation pattern within this lesion had changed in a similar fashion to the anterior one. Fluorodeoxyglucose (FDG) positron emission tomography (PET) demonstrated slightly increased uptakes in the anterior and posterior mediastinal lesions, with maximum standardized uptake values of 2.5 (Figure 3) and 1.6 in the late phase, respectively.

**FIGURE 1:**
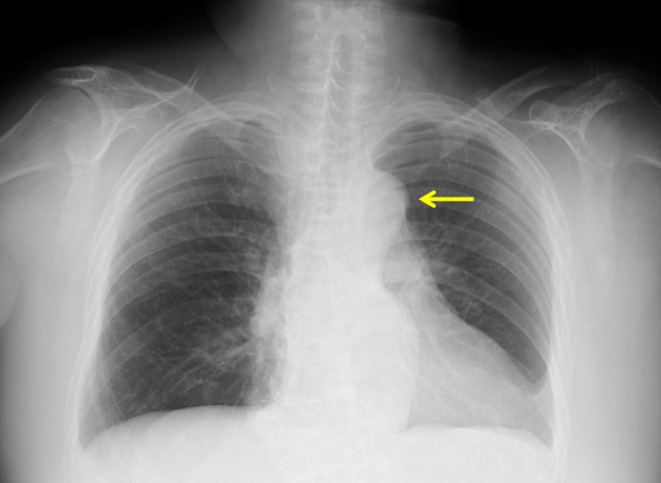
A chest X‐ray revealed a slight prominence of the superior mediastinum, projecting to the left (arrow).

**FIGURE 2:**
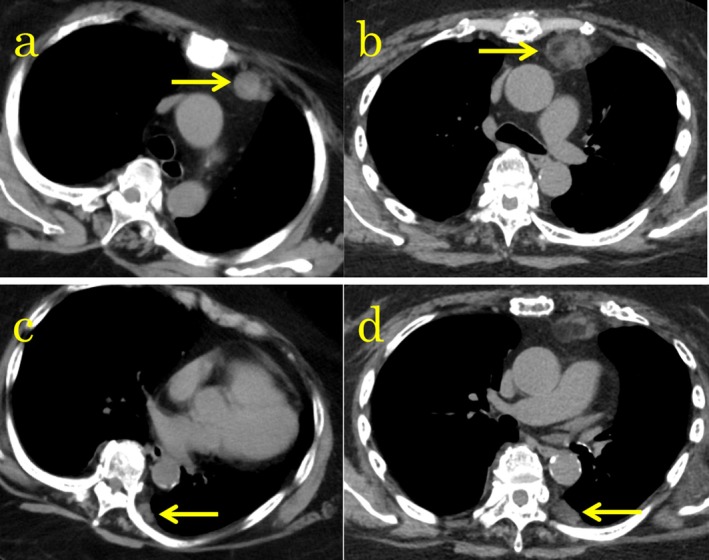
Unenhanced chest computed tomography (CT) showed a well‐demarcated anterior mediastinal lesion located behind the sternum at the left second costochondral junction, which had increased in size from 2.6 cm (a) to 3.7 cm (b) over 4 years (arrows). The CT attenuation pattern within the lesion had also changed over time, shifting from a predominantly soft tissue density to a heterogeneous composition of soft tissue and fat. Additionally, a small lesion was detected in the posterior mediastinum at the left seventh intercostal space, which had enlarged from 1.5 cm (c) to 2.0 cm (d) over the same period (arrows).

**FIGURE 3:**
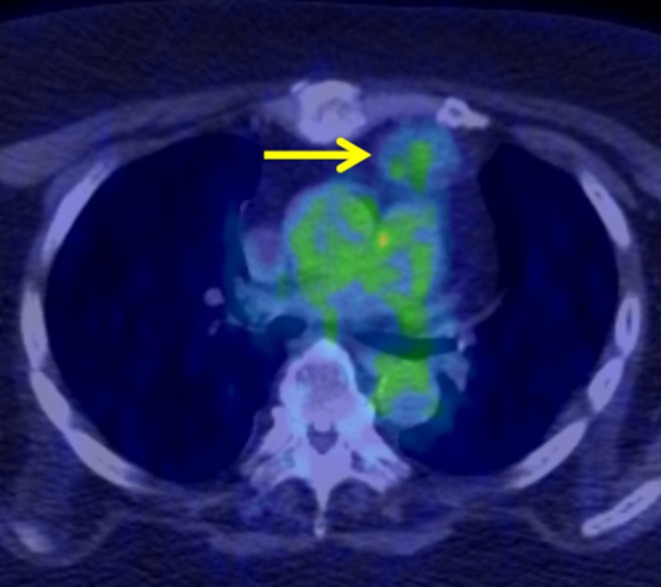
Fluorodeoxyglucose positron emission tomography revealed a slightly increased uptake in the anterior mediastinal lesion, with a maximum standardized uptake value of 2.5 in the late phase (arrow).

## 3 Differential Diagnosis, Investigations and Treatment

Based on these radiological findings and the lesions' slow growth rates, a presumptive diagnosis of thymoma with possible pleural seeding, thymolipoma, teratoma, or neurogenic tumor was made.

After obtaining the patient's consent, we proceeded with excisional resection of both lesions via thoracotomy for definitive diagnosis and treatment. Intraoperatively, the anterior lesion was found to be vascularized by branches of the internal mammary vessels, while the posterior lesion was supplied by the intercostal artery, indicating their hypervascular nature. Both lesions were successfully excised with minimal blood loss.

## 4 Outcome and Follow‐Up

The chest drain was removed on postoperative day (POD) three, and the patient recovered uneventfully. She was discharged on POD seven.

Macroscopically, the resected specimens appeared dark‐red and yellow. Histological examination revealed trilineage hematopoietic elements, including erythrocytes, granulocytes, and megakaryocytes, interspersed with mature adipose tissue (Figure 4). Based on these findings, a diagnosis of EMH was confirmed.

**FIGURE 4:**
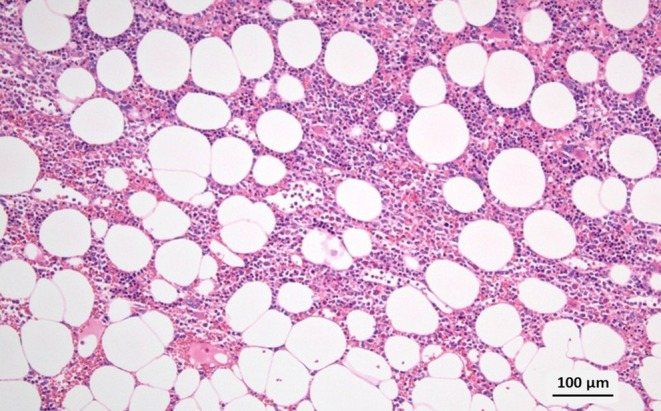
Histological examination revealed trilineage hematopoietic elements, including erythrocytes, granulocytes, and megakaryocytes, interspersed with mature adipose tissue (hematoxylin–eosin staining).

A peripheral blood smear, reticulocyte count, and iron studies did not reveal evidence of occult ineffective erythropoiesis. Although further hematologic evaluation was warranted, a bone marrow biopsy was not performed due to the patient's significant comorbidities. She has been relapse‐free for 14 months since the resection.

## 5 Discussion

Intrathoracic EMH is well known to predominantly occur in the posteroinferior mediastinum. One proposed explanation for this location is the extrusion of marrow through the thin cortex of the vertebral bodies, a phenomenon often observed in patients with hemoglobinopathies [2]. The present case is considered extremely rare, as EMH arose in the anterior mediastinum without any underlying hematological disorders.

To our knowledge, this is the third described case of anterior mediastinal EMH mimicking thymic tumors. Catinella et al. reported the first case in a 68‐year‐old obese man [3], where a definitive diagnosis was established via median sternotomy and tumor excision. Santini et al. reported a second case involving a 57‐year‐old obese woman [4], in whom a definitive diagnosis was confirmed through a video‐assisted thoracoscopic approach and tumor biopsy. In the present case, a definitive diagnosis was established following tumor excision via thoracotomy. Table 1 shows the clinicoradiological features of reported cases of anterior mediastinal EMH. Intriguingly, all three cases shared the characteristics of obesity. Myelolipoma, a disease that shows histological similarity to EMH, is known to have a close relationship with obesity [5, 6]. Anterior mediastinal EMH may also be associated with obesity.

**TABLE 1:** EMH in the anterior mediastinum.

Author	Year	Age	Sex	Obesity	Hematologic disorder	CT finding
Catinella et al. [3]	1985	68	M	+	None	Heterogeneous
Santini et al. [4]	2009	57	F	+	None	Homogeneous
Present case	2026	67	F	+	None	Homogeneous → Heterogeneous (fat admixture over time)

FDG‐PET finding	Approach	Surgical procedure	Postoperative complication
Not performed	Median sternotomy	Excision	None
Increased uptake	VATS	Biopsy	None
Slight uptake	Thoracotomy	Excision	None

Abbreviations: CT, computed tomography; EMH, extramedullary hematopoiesis; FDG, fluorodeoxyglucose; PET, positron emission tomography; VATS, video‐assisted thoracoscopic surgery.

The computed tomographic features of EMH lack specificity [7]. Hematopoietic elements exhibit high attenuation, similar to soft tissue, whereas fat tissue shows low attenuation, resulting in a heterogeneous appearance depending on the varying proportions of hematopoietic and fat tissue components. In the present case, the CT attenuation of the anterior mediastinal EMH changed over 4 years from a relatively homogeneous soft tissue density to a heterogeneous density. This transformation likely reflects the gradual infiltration of fat tissue within the EMH lesion. When an anterior mediastinal lesion shows a slow growth rate and fat admixture on CT, differential diagnosis should include EMH as well as thymoma, thymolipoma, and teratoma.

FDG‐PET findings in EMH have rarely been reported. Although previous reports demonstrated markedly increased uptake [2, 4], our case had only slightly increased uptake. This discrepancy may also be attributable to the different proportions of hematopoietic elements and fat tissue within the EMH lesions. Further research on FDG accumulation in EMH lesions is needed.

Other noninvasive methods to establish the diagnosis of intrathoracic EMH are bone marrow scintigraphies using In‐111 chloride and Tc‐99 m Sn colloid [8]. These studies may obviate the need for invasive methods such as needle biopsy or surgical excision, reducing the risk of hemorrhagic complications. Although EMH masses are typically vascular, we believe that biopsy/invasive procedures should be performed to confirm definitive diagnosis and guide management [1].

In conclusion, we report an extremely rare case of intrathoracic EMH arising in the anterior mediastinum. EMH should be considered in the differential diagnosis of anterior mediastinal lesions, particularly when CT shows a slow growth rate and evolving fat content. Although EMH masses may be hypervascular and various imaging modalities are useful for the diagnosis of EMH, histologic confirmation is desirable to obtain a definitive diagnosis and guide management.

## Author Contributions

**Yoshiki Kozu:** conceptualization, writing – original draft. **Tomoaki Kinno:** writing – review and editing. **Kazunori Kajino:** investigation, writing – review and editing. **Kenji Suzuki:** supervision, writing – review and editing.

## Funding

The authors have nothing to report.

## Ethics Statement

Our institutional ethics committee granted that no ethical approval was required.

## Consent

Written informed consent was obtained from the patient for publication of this case report and accompanying images.

## Conflicts of Interest

The authors declare no conflicts of interest.

## Data Availability

The data supporting the findings of this study are available within the article.
